# High dietary cholesterol and ovariectomy in rats repress gene expression of key markers of VLDL and bile acid metabolism in liver

**DOI:** 10.1186/s12944-015-0128-9

**Published:** 2015-10-09

**Authors:** Zahra Farahnak, Isabelle Côté, Emilienne T. Ngo Sock, Jean-Marc Lavoie

**Affiliations:** Département de Kinésiologie, Université de Montréal, C.P. 6128, Succ. centre-ville, Montréal, Québec H3C 3 J7 Canada

**Keywords:** Liver cholesterol, Cholesterol diet, VLDL synthesis, LDL receptors

## Abstract

**Background:**

The purpose of the study was to evaluate the effects of high dietary cholesterol in ovariectomized (Ovx) rats on several key markers of hepatic cholesterol and bile acid metabolism.

**Method:**

Ovx and sham operated (Sham) rats were given either a standard diet (SD), a SD diet supplemented with 0.25 % cholesterol (SD + Chol), or a high fat diet supplemented with 0.25 % cholesterol (HF + Chol) for 5 weeks.

**Results:**

Ovx was associated with higher (*P* < 0.05) liver total cholesterol (TC) under the SD and the SD + Chol diet, while liver triglyceride (TG) content was higher in Ovx than in Sham rats in all 3 diet conditions. Surprisingly, the SD + Chol diet was associated with lower (*P* < 0.001) plasma TC and TG levels in Ovx than in Sham rats, suggesting a decrease in VLDL secretion. Accordingly, several transcripts of key markers of VLDL synthesis including microsomal TG transfer protein (*Mttp*) and *Apob-100* were decreased (*P* < 0.05) in Ovx compared to Sham rats under the three dietary conditions and even more so for *Mttp* and *Apob-100* when rats were fed the SD + Chol diet. Transcripts of bile acid transporters including bile salt export pump (*Bsep*) and Na + −taurocholate cotransporting polypeptide (*Ntcp*) were decreased by the addition of cholesterol to the SD diet in both Ovx and Sham rats.

**Conclusion:**

These results indicate that a high cholesterol feeding and ovariectomy combine to reduce the gene expression of key markers of VLDL synthesis suggesting a reduction in excretion of cholesterol from the liver.

## Introduction

There has been accumulating evidence in recent years that estrogens deficient state in ovariectomized (Ovx) animals and in postmenopausal women results in substantial liver triglyceride (TG) accumulation [1–4]. On the other hand, evidence of disturbances of cholesterol metabolism in link with estrogens deficiency is limited to observations of higher plasma levels of total cholesterol found in human as well as in animal models [5–8]. Liver cholesterol metabolism in Ovx animals has received little attention and shows some controversies. For instance, liver total cholesterol (TC) level was reported not to be affected by Ovx in previous studies [9, 10] although we recently found an increase in rats ovariectomized for 8 wks [11]. There is, therefore, a need for more physiological and molecular information to better understand how liver, as a master regulator of cholesterol metabolism, is affected by estrogens withdrawal.

Nutritional approaches have been used frequently as a tool to investigate the role of the liver in regulating TG and cholesterol metabolism [12, 13]. In Ovx animals, our group recently observed a large cholesterol accumulation in liver of rats fed a high fat (HF) diet, suggesting a vulnerability to cholesterol accumulation of Ovx animals when fed a HF diet [14]. The vulnerability of Ovx animals to dietary cholesterol has also been recently enlighten by the demonstration that gene expression of several molecular markers of VLDL assembly were reduced following high fat/high cholesterol diets [15]. However, dietary fat and dietary cholesterol have been reported to result in a positive synergistic interaction on the development of for instance hypercholesterolemia [13]. We, therefore, postulated that a better understanding of how Ovx animals regulate hepatic cholesterol metabolism would be obtained if the animals were fed a high cholesterol diet without the confounding effect of dietary fat.

In an attempt to shed some light on how liver of Ovx animals respond to high dietary cholesterol, we targeted key molecular markers of pathways involved in cholesterol/bile acids metabolism/transport that have recently been found to be affected by estrogens deficiency [15]. We first looked at molecular markers of VLDL assembly, including microsomal TG transfer protein (*Mttp*), a rate limiting molecule in VLDL assembly and secretion, *Apob-100* an essential structural protein that translocates into the luminal side of the endoplasmic reticulum, diacylglycerol acyltransferase 2 (*Dgat2*) involved in converting fatty acids into triglyceride (TG), acyl-Coa: cholesterol acyl transferase (*Acat*2) that converts free cholesterol into cholesterol ester, cell death-inducing like-effector type B (*Cideb*) a protein involved in lipidation of particles, and small GTP-binding protein a (Sar1a) a protein that facilitates the movements of VLDL particles toward the Golgi apparatus.

Furthermore, we investigated the gene expression of molecular markers of bile acids metabolism/transport, a pathway that is tightly associated with elimination of cholesterol from the liver. These included ATP-cassette binding protein G5 and G8 (*Abcg5/Abcg8*) that export cholesterol from hepatocytes to the bile duct, bile salt export pump (*Bsep*) and multidrug resistance-associated transporter 2 (*Mdr2*) which stimulate bile acid and phospholipid transport from hepatocytes to bile canaliculi, Na + −taurocholate cotransporting polypeptide (*Ntcp*) involved in bile acid uptake in the basolateral membrane of the hepatocytes, farnasoid X receptor (*Fxr*) a nuclear receptor involved in regulation of hepatic bile acid biosynthesis, and cytochrome P450 7A1 (*Cyp7a1*) the main enzyme that catalyses the conversion of cholesterol into bile acids. Finally, we complemented our approach by investigating the response of hepatic LDL-receptor (*Ldl-r*), a major determinant of removal of LDL-cholesterol particles from the circulation, LDL receptor-related protein-1 (*Lrp-1*) involved in the removal of plasma remnant lipoproteins [16, 17], and sterol regulatory element-binding protein-2 (*Srebp2*) a transcription factor involved in the regulation of cholesterol.

The aim of the present study was to determine the effects of high dietary cholesterol on hepatic key markers of VLDL and cholesterol/bile acid metabolism in Ovx rats.

## Methods

### Animal care

Female Sprague–Dawley rats (*n* 48; Charles River, St Constant, PQ, Canada) weighing 180–200 g upon arrival were housed individually. Food and water were supplied *ad libitum.* Their environment was controlled in terms of light (12 h light–dark cycle starting at 06:00 AM), humidity and room temperature (20–23 °C). Body weight and food intake were monitored two times per week. All experimental procedures were conducted according to the protocols approved by the directives of the Canadian Council on Animal Care after institutional approval.

### Diets and surgery

Rats were first acclimated to their environment for a period of one week while fed a chow diet (12.5 % lipid, 63.2 % CHO and 24.3 % protein; kJ from Agribrands Canada, Woodstock, Ontario, Canada). Thereafter, rats underwent either a bilateral ovariectomy (Ovx, *n* 24) or a bilateral sham-operation (Sham, *n* 24) according to the technique described by Robertson *et al.* under isoflurane anaesthesia [18]. After surgery, animals were injected with antibiotics (Tribrissen 24 %; 0.125 cc/kg, subcutaneously) and analgesic (Carprofen; 4.4 mg/kg, subcutaneously) for 3 days. Thereafter, the Ovx and Sham rats were assigned to one of three following diets: standard (SD), SD supplemented with 0.25 % cholesterol (SD + Chol), or high fat supplemented with 0.25 % cholesterol (HF + Chol) for 5 weeks (Table 1). The 0.25 % cholesterol dose was chosen to increase the dietary cholesterol without the atherogenic effects of higher doses used in several other studies [12, 13].

**Table 1:** Diet description

	Standard diet (SD) (D12450J)	SD + Chol (0.25 %) (D13020701)	High Fat + Chol (0.25 %) (D13020703)
(%)			
Protein	19.2	19.2	22.8
Carbohydrate	67.3	67.3	45.7
Fat	4.3	4.3	20.2
(g)			
Casein	200	200	200
L-Cysine	3	3	3
Corn Starch	506.2	506.2	202.5
Maltodextrin 10	125	125	125
Sucrose	68.8	68.8	68.8
Cellulose, BW200	50	505	50
Soybean oil	25	25	25
Lard	20	20	155
Mineral Mix S10026	10	10	10
DiCalcium Phosphate	13	13	13
Calcium carbonate	5.5	5.5	5.5
Potassium citrate, 1 H2O	16.5	16.5	16.5
Vitamin mix V10001	10	10	10
Choline bitartrate	2	2	2
Cholesterol	0.0	2.63	2.63
Kcal/g	3.85	3.84	4.56

Formulateds by: Research Diets, Inc. (20 Jules Lane, New Brunswick, NJ 08901 USA)

### Blood and tissue sampling

Rats were fasted overnight and sacrificed between 09:00 and 12:00 AM. Immediately after complete anaesthesia with isoflurane, the abdominal cavity was opened following the median line of the abdomen and approximately 4 ml of blood was collected from the abdominal vena cava (<45 s) into syringes pre-treated with ethylenediaminetetraacetic acid (15 %; EDTA). Blood was centrifuged (3000 rpm; 4 °C; 10 min; Beckman GPR Centrifuge) and the plasma kept for further analyses. Immediately after blood collection, the liver median lobe was removed and freeze-clamped. This sample was used for triacylglycerol (TG), cholesterol, and mRNA determinations. Several organs and tissues were removed and weighed (Mettler AE 100) in the following order: uterus, mesenteric, urogenital, and retroperitoneal fat deposits. The mesenteric fat pad consisted of adipose tissue surrounding the gastrointestinal tract from the gastroesophageal sphincter to the end of the rectum. The urogenital fat pad included adipose tissue surrounding the kidneys, uterus and bladder as well as ovaries, oviducts and uterus. The retroperitoneal fat pad was taken as that distinct deposit behind each kidney along the lumbar muscles. All tissue samples were frozen in liquid nitrogen immediately after being weighed (Mettler AE-100). All tissue samples were stored along with plasma samples at −80 °C until analyses were performed.

### Biochemical analyses

Commercial kits from Sigma (Sigma; St-Louis, Missouri, USA) were used to determine plasma and liver TG by colorimetric method. Liver TG concentrations were estimated from glycerol released after KOH hydrolysis. Total liver cholesterol concentrations were determined with some adaptations of the procedure described by Folch *et al.* [19]. Briefly, 0.1 g of liver was homogenized with chloroform–methanol mixture (2:1, v/v). The chloroform layer was collected and evaporated overnight. After adding 10 % Triton X-100 in isopropanol, the sample was assayed for total cholesterol using commercial kits according to the manufacturer’s instructions (Wako Diagnostics and Chemicals USA, Richmond, VA, USA). Plasma total cholesterol was determined using the same kits supplied by Wako.

### Molecular analyses

Total RNA was extracted from frozen liver with the use of RNA extraction Mini kit (Invitrogen) according to the manufacturer’s protocol. Then RNA was treated with DNase (Invitrogen) in order to avoid genomic contamination. Total RNA (2 μg) was reverse-transcribed into complementary DNA using high capacity complementary DNA reverse transcription kits (Applied Biosystems). RT samples were stored at −20 °C. Gene expression for *β-actin* was determined using a pre-validated Taqman Gene Expression Assay (Applied Biosystems, Rn0146*26*61, Foster City, CA). Gene expression level for target genes was determined using assays designed with the Universal Probe Library from Roche. The primer sets and UPL probe numbers are presented in Table 2. To validate the efficiency of the qPCR assays, we used a mix of the samples tested in the study.

**Table 2:** Oligonucleotide primers used for quantitative real-time polymerase chain reaction

Gene	Oligo FWD	Oligo REV
MDR2	ggcattctccatcatcctgt	cacttctgttgctttactgtgtca
BSEP	cggtggctgagagatcaaat	tgcgatagtggtggagaaca
ABCG5	cggagagttggtgttctgtg	caccgatgtcaagtccatgt
ABCG8	cagatgctggctatcataggg	ctgatttcatcttgccacca
ACAT-2	cctcacagatgcgtttcaca	ctctgctcacttgccattttt
Apob	gatggagatgggagatgaggt	gggctcctcatcaacaagag
Cideb	gctccaatggcctgctaag	ttatgatcacagacacggaagg
Cyp7a1	ggagcttatttcaaatgatcagg	cactctgtaaagctccactcactt
DGAT-2	aggatctgccctgtcacg	gtcttggagggccgagag
HMG-CoAr	caaccttctacctcagcaagc	acagtgccacacacaattcg
LDLr	tgctactggccaaggacat	ctgggtggtcggtacagtg
LRP-1	aatcgagggcaagatgacac	ccagtctgtccagtacatccac
Mttp	gcgagtctaaaacccgagtg	cactgtgatgtcgctggttatt
FXR	ccacgaccaagctatgcag	tctctgtttgctgtatgagtcca
Sar1a	gggcaaaccacaggaaag	cactgcacatgaacacttcca
SREBP-2	gtgcagacagtcgctacacc	aatctgaggctgaaccagga
NTCP	aaaatcaagcctccaaaggac	ttgtgggtacctttttccaga
ActB	cccgcgagtacaaccttct	cgtcatccatggcgaact
GAPDH	ccctcaagattgtcagcaatg	agttgtcatggatgaccttgg

The ABI PRISM® 7900HT (Applied Biosystems) was used to detect the amplification level and was programmed with an initial step of 3 min at 95 °C, followed by 40 cycles for 5 s at 95 °C and 30 s at 60 °C. All reactions were run in triplicate and the average values of threshold cycle *(C*_T_*)* were used for quantification. *β-actin* was used as endogenous control. The relative quantification of target genes was determined using the ∆∆*C*_T_ method. Briefly, the *C*_T_ values of target genes were normalized to an endogenous control gene (*β-actin*) (∆*C*_T_ = *C*_T__target_ – *C*_T__β-actin_) and compared with a calibrator: (∆∆ *C*_T_ = ∆ *C*_T__Sample_ - ∆*C*_T__Calibrator_). Relative expression (RQ) was calculated using the Sequence Detection System (SDS) 2.2.2 software (Applied Biosystems) and the formula is RQ = 2^-∆∆*C*^_T_.

### Statistical analysis

All data are presented as mean ± SE. Statistical significance *(P* < 0.05*)* was determined using a 2-way ANOVA for non-repeated measures with ovariectomy and diets as main factors. Fisher LSD *post hoc* test was used in the event of a significant interaction effect. For a significant diet effect without interaction, Fisher LSD from a one-way ANOVA was used.

## Results

There was no difference in initial body weights for the 6 groups (means ± SE: 190.6 ± 1.9 to 193.3 ± 1.7). Body weight, intra-abdominal fat pad weight and food intake measured at the end of the experiment were all significantly (*P* < 0.01) higher in Ovx than in Sham animals in the three dietary groups (Table 3). The addition of Chol to the SD diet did not result in any changes in both Ovx and Sham rats in body weight, intra-abdominal fat pad weight, and food intake when compared to animals fed the SD diet. However, food intake was significantly (*P* < 0.05) higher in rats fed the HF + Chol diet as compared to the two other diets in both Sham and Ovx animals. Body weight and intra-abdominal fat pad weight also showed a strong tendency to be higher in rats fed the HF + Chol diet with respect to the Sham and Ovx conditions (*P* = 0.06 and *P* = 0.08, respectively) . Uterus weight was lower (*P* < 0.001) in Ovx than in Sham rats throughout the dietary conditions confirming total ovariectomy (Table 3).

**Table 3:** Anthropometric parameters and food intake

Variables	SD		SD + Chol (0.25 %)		HF + Chol (0.25 %)	
	Sham	Ovx	Sham	Ovx	Sham	Ovx
Final body weight (g)	353.9 ± 11.3	439.6 ± 14.5***	347.5 ± 8.7	431.6 ± 25.7***	391.6 ± 13.7	467.4 ± 15.6***
Intra-abdominal fat pad weights (g)	29.2 ± 4.4	39.3 ± 3.7**	26.6 ± 2.6	40.2 ± 4.9**	38.7 ± 3.8	43.8 ± 2.6**
Food intake (kcal/day)	82.7 ± 4.3	99.8 ± 4.2***	81 ± 2.5	95.7 ± 7.0***	94.9 ± 4.7^†δ^	110.1 ± 4.2***^†δ^
Uterus (g)	0.50 ± 0.03	0.10 ± 0.01***	0.51 ± 0.07	0.10 ± 0.01***	0.47 ± 0.05	0.11 ± 0.01***

SD standard diet, HF + Chol (0.25 %) high fat + cholesterol, *Ovx* ovariectomised, *Sham* sham operated

Values are mean ± SE with *n* = 8 rats per group

**Significantly different from the sham groups (*P* < 0.01), ***(*P* < 0.001)

^†^Significantly different from SD diet (*P* < 0.05)

^δ^Significantly different from SD+ Chol diet (*P* < 0.05)

### Plasma and liver lipid profile

**Fig 1:**
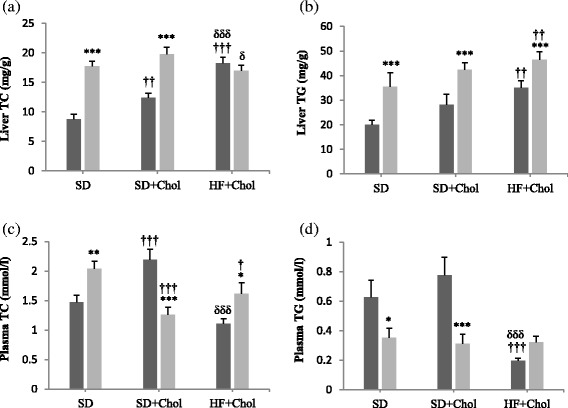
Liver and plasma total cholesterol (TC) and triacylglycerol (TG) levels in sham (■) and ovariectomized (Ovx, gray square symbol) rats fed a standard diet (SD), a SD + 0.25 % cholesterol diet (SD + Chol), and a high fat + 0.25 % cholesterol diet (HF + Chol). Values are mean ± SE with *n* = 8 rats per group. *Significantly different from respective Sham rats (*P* < 0.05), **(*P* < 0.01), ***(*P* < 0.001); ^†^Significantly different from respective rats fed the SD diet (*P* < 0.05), ^††^(*P* < 0.01), ^†††^(*P* < 0.001); ^δ^Significantly different from respective rats fed the SD + Chol diet (*P* < 0.05), ^δδδ^(*P* < 0.001)

Liver TC levels in Sham rats were progressively higher (*P* < 0.01) following the SD, SD + Chol and HF + Chol diets order (Fig. 1(a)). This was not observed, however, in Ovx animals. Liver TC levels were on the whole higher (*P* < 0.001) in Ovx than in Sham rats but not under the HF + Chol diet (Fig. 1(a)). Liver TG levels were higher (*P* < 0.001) in Ovx than in Sham rats in all three dietary conditions (Fig. 1(b)). Liver TG levels were not affected by the SD + Chol diet while it was higher (*P* < 0.01) in the HF + Chol diet compared to the SD diet in both Ovx and Sham animals. A completely different picture was observed for the TC and TG concentrations measured in plasma. Plasma TC levels were higher (*P* < 0.05) in Ovx than in Sham rats under the SD and HF + Chol diets but largely lower (*P* < 0.001) in Ovx than in Sham animals under the SD + Chol diet (Fig. 1(c)). On the whole, plasma TC levels in Ovx rats were lower (*P* < 0.05) under the two Chol diets as compared to the SD diet. On the opposite of liver TG, plasma TG levels were lower (*P* < 0.05) in Ovx than in Sham rats under the SD and SD + Chol diets (Fig. 1(d)). Plasma TG values were also lower (*P* < 0.001) in Sham rats fed the HF + Chol diet as compared to Sham rats in the 2 other diets. On the whole, TC and TG levels under the SD + Chol feeding were higher in liver of Ovx vs Sham animals while the opposite was found in plasma.

### Hepatic gene expression

**Fig 2:**
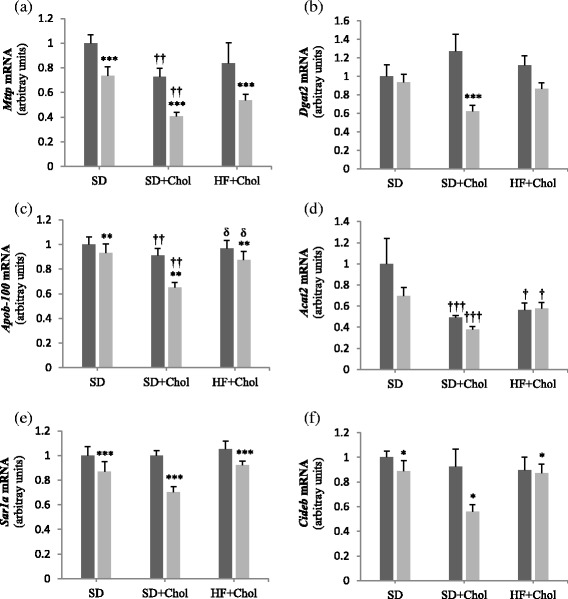
Hepatic mRNA expression of genes involved in VLDL synthesis/production in sham (■) and ovariectomized (Ovx, gray square symbol) rats fed a standard diet (SD), a SD + 0.25 % cholesterol diet (SD + Chol), and a high fat + 0.25 % cholesterol diet (HF + Chol). Values are mean ± SE with *n* = 8 rats per group. ***Significantly different from respective Sham rats (*P* < 0.001); ^†^Significantly different from rats fed the respective SD diet (*P* < 0.05), ^††^(*P* < 0.01), ^†††^(*P* < 0.001); ^δδ^Significantly different from rats fed the respective SD + Chol diet (*P* < 0.01). *Mttp* microsomal TG transfer protein; *Dgat-2*, diacylglycerol acyl transferase-2; *apoB-100*, apolipoprotein B-100; *Acat-2,* acyl-coA cholesterol acyl transferase-2; *Sar1a,* small GTP-binding protein; *Cideb*, cell death-inducing like-effector type B

Gene expressions of key molecules involved in VLDL synthesis are presented in Fig. 2. On the whole, mRNA levels of 5 out of the 6 genes involved in VLDL synthesis (including *Sar1a* and *Cideb* and with the exception of *Acat2*) were lower (*P* < 0.05) in Ovx than in Sham rats in all dietary conditions (only under the SD + Chol diet for *Dgat2*). The lowest (*P* < 0.01) values for *Mttp*, *Dgat2,* and *Apob-100* transcripts were found in Ovx rats under the SD + Chol (as compared to the SD diet), suggesting that Ovx and the SD + Chol diet combine to decrease VLDL synthesis. Interestingly, we found that the decreased responses in gene expression of *Mttp*, *Dgat2*, *Acat2*, and *Apob-100* under the SD + Chol diet were statistically attenuated (as compared to the SD diet) when rats were fed the HF + Chol diet (Fig. 2).

**Fig. 3:**
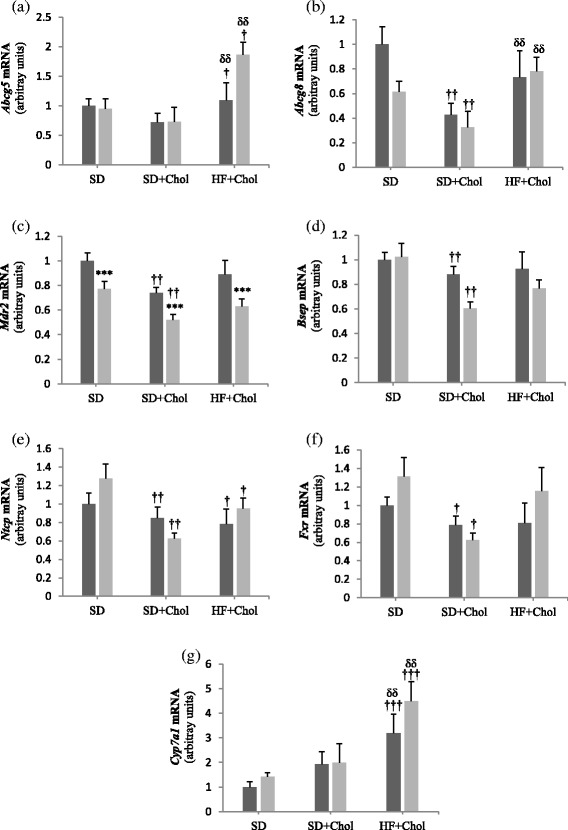
Hepatic mRNA expression of genes related to bile acid metabolism in sham (■) and ovariectomized (Ovx, gray square symbol) rats fed a standard diet (SD), a SD + 0.25 % cholesterol diet (SD + Chol), and a high fat + 0.25 % cholesterol diet (HF + Chol). Values are mean ± SE with *n* = 8 rats per group. ***Significantly different from respective Sham rats (*P* < 0.001); ^†^Significantly different from rats fed the respective SD diet (*P* < 0.05), ^††^(*P* < 0.01), ^†††^(*P* < 0.001); ^δδ^Significantly different from rats fed the respective SD + Chol diet (*P* < 0.01), ^δδδ^(*P* < 0.001). *Abcg5/Abcg8*, ATP-cassette binding protein G5 and G8; *Mdr2*, multidrug resistance-associated transporter 2; *Bsep*, bile salt export pump; N*tcp*, Na + −taurocholate cotransporting polypeptide; *Cyp7a1*, cytochrome P450 7A1; *Fxr*, farnesoid X receptor

To complete the information we investigated genes involved in hepatic cholesterol and biliary acids transport (Fig. 3). With the exception of *Mdr2* transcripts, we found no difference between Ovx and Sham rats in any of the measured gene expression in all dietary conditions (Fig. 3). However, we observed that several of the genes (*Abcg8*, *Mdr2*, *Bsep*, *Fxr,* and *Ntcp*) had their transcripts decreased when animals were fed the SD + Chol compared to the SD diet. On the other hand, *Abcg5/g8* and *Cyp7a1* mRNA levels were higher (*P* < 0.01) in HF + Chol compared to SD + Chol fed animals.

**Fig. 4:**
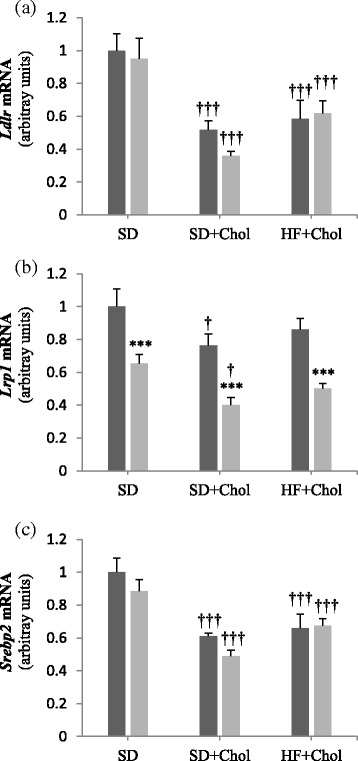
Hepatic mRNA expression of genes involved in uptake of cholesterol rich lipoproteins from the circulation in sham (■) and ovariectomized (Ovx, gray square symbol) rats fed a standard diet (SD), a SD + 0.25 % cholesterol diet (SD + Chol), and a high fat + 0.25 % cholesterol diet (HF + Chol). Values are mean ± SE with *n* = 8 rats per group. ***Significantly different from respective Sham rats (*P* < 0.001); ^†^Significantly different from rats fed the respective SD diet (*P* < 0.05), ^†††^(*P < 0.001*). *Ldlr*, LDL-receptor; *Lrp-1,* LDL receptor-related protein-1; *Srebp2,* sterol regulatory element-binding protein-2

To further explore hepatic cholesterol metabolism, we measured gene transcripts of key molecules involved in cholesterol uptake from cholesterol rich lipoproteins. We first found a lower (*P* < 0.001) *Lrp1* gene expression in Ovx than in Sham animals in all dietary groups (Fig. 4(b)). Interestingly, we observed that gene expression of *Ldlr* and *Srebp2* were highly decreased (*P* < 0.001) when rats were fed either the SD + Chol or the HF + Chol diets as compared to rats fed the SD di et, while *Lrp1* transcripts were decreased (*P* < 0.05) only under the SD + Chol diet (Fig. 4).

## Discussion

The main finding of the present study is that high dietary cholesterol represses gene expression of key molecular markers of VLDL synthesis (*Mttp, Acat2, Apob-100*) in Sham rats and even more so in Ovx rats (*Mttp* and *Apob-100*). In addition, the sole addition of cholesterol to the SD diet reduced gene expression of several liver markers of bile acid and phospholipid transport (*Bsep, Ntcp, Mdr2*) in both Sham and Ovx animals. Finally, gene expression of key markers involved in liver LDL cholesterol uptake (*Ldl-r* and *Lrp1*) was also decreased by the sole addition of cholesterol to the diet in Sham as well as in Ovx rats. These results first indicate that the cholesterol component in a mixed diet is a determinant factor that regulates liver cholesterol metabolism in sham as well as in Ovx rats. In addition, the present molecular responses to high cholesterol diet converge to indicate a reduction in hepatic TG and cholesterol excretion from the liver, this being to a certain extent, accentuated by the absence of estrogens.

We recently reported data showing an impairment of VLDL assembly following ovariectomy and high fat/high cholesterol diets in rats [15]. The present study extends these findings by indicating that the sole high *content* of cholesterol in the diet impaired VLDL assembly in Sham and even more so in Ovx animals. Ovx has been previously associated with a decrease in VLDL production in rat *via Mttp* regulation [20]. Accordingly, molecular expression of several genes related to VLDL assembly, including *Mttp* and *Apob*, was reduced in Ovx rats under the present SD diet. That high cholesterol diet decreases even more so VLDL assembly/production in Ovx animals suggests an additive effect of these two stimuli. This additive effect on VLDL assembly/production is corroborated by the higher accumulation of TC and TG in liver along with lower levels of plasma TC and TG found in Ovx compared to Sham animals under the SD + Chol diet.

It is not clear at this point the mechanism by which a high cholesterol diet would repress VLDL assembly/production. *Hepatic* VLDL production has been reported to be reduced by competitive inhibitors of HMG-CoA reductase, the main enzyme responsible for cholesterol biosynthesis, through the regulation of the SREBP family transcription factors [21, 22]. SREBP-2 gene expression was repressed by the cholesterol diet in the present study. Although there is evidence that several steps of the VLDL assembly/secretion are under the control of SREBP-1, there are also indications that upon the cell type and physiological conditions, SREBP1 and SREBP2 may mediate changes in lipoprotein assembly and secretion [22]. An alternative explanation for the decrease in VLDL synthesis/production following high dietary cholesterol and Ovx would be the endoplasmic reticulum (ER) stress. There are indications that cholesterol can induce hepatic ER stress through free cholesterol accumulation in the ER [23], and that ER stress limits VLDL assembly and secretion through apoB degradation [24].

In addition of reducing VLDL synthesis/production, high cholesterol feeding in Ovx and Sham animals also repressed gene expression of key markers of bile acid metabolism. The present finding of a reduction in *Bsep* and *Mdr2* gene expression suggests a reduction in bile acid and phospholipid excretion, while the reduction in *Ntcp* mRNA suggests a reduction in bile acid uptake from the entero-hepatic circulation. These observations may be taken as an indication of a reduction of the entero-hepatic circulation of bile acids. Furthermore, the reduction in *Fxr* gene expression in the SD + Chol fed rats suggests that even though cholesterol level was increased in liver, there was no accumulation of bile acids since the role of hepatic *Fxr* is to prevent bile acid hepatotoxicity. Dietary interventions such as high cholesterol/high fat diets have been reported to repress *Fxr* gene expression in liver [13, 15]. The gene expression of the present key molecules thus supports the previous suggestion that high cholesterol feeding in rats disrupts bile acid metabolism [15].

In addition to a decrease in gene expression of markers of VLDL synthesis and bile acid transport, dietary cholesterol in the present study resulted in a down regulation in gene expression of *Ldl-r* and *Lrp1* in Sham and Ovx animals suggesting a decrease in cholesterol uptake from circulation. This response was even more pronounced in Ovx rats for *Lrp1* transcripts. Ovx has been previously reported to be associated with a reduction in the expression of several genes involved in the uptake of lipoprotein molecules [11, 25]. The decrease in LDL receptors was most likely linked to the excess hepatic cholesterol level through the decrease in *Srebp2* gene expression [26, 27]. Taken together, the present results suggest an association between reduced VLDL synthesis/production, reduced bile acids transport and reduced LDL receptors under high dietary cholesterol in Sham as well as in Ovx rat.

The interest of comparing a high cholesterol diet with and without the addition of a high fat content is enlighten by the observation that the sole addition of cholesterol to a SD diet had no effect on body weight in Sham and Ovx animals while the addition of fat in the diet caused an increase in food intake and a strong tendency in higher body weight (*P* = 0.06) and intra-abdominal (*P* = 0.08) fat accumulation (Table 3). This implies, on a clinical point of view, that a high cholesterol diet might not be perceived as being deleterious since it does not affect body weight when in fact it causes several metabolic perturbations. One noticeable effect of adding fat to cholesterol in the diet was the increase in liver fat accumulation resulting from the diet and most likely from increased lipogenesis [28]. On the other hand, higher expression of *Abcg5/g8* and *Cyp7a1* were also observed under HF + Chol feeding, both of these genes being involved in cholesterol excretion from the liver. These responses may be taken as an indication that hepatic cholesterol metabolism may be less vulnerable to high fat/high cholesterol feeding than high dietary cholesterol alone.

The present results have limitations that need to be taken into consideration. First, the limited duration (5 weeks) of the present experimental conditions might represent an acute situation that may lead to different results on a longer term basis with a chronic adaptation to the diet. The present results generated from an animal model are intended to open research issues that need to be tested in humans.

In summary, results of the present study first indicate that gene expressions of key markers of VLDL synthesis/production are reduced under high cholesterol feeding and that this reduction is exacerbated in Ovx animals. In addition, the present data provide evidence that the activities of bile acid and *Ldl-r* pathways are also reduced by the sole addition of cholesterol to a SD diet in Sham as well as in Ovx animals. These results point to the direction as if the liver under high cholesterol feeding reduces its excretion of cholesterol, at least on a short term basis, thus contributing to exacerbate liver fat and cholesterol accumulation known to occur in Ovx animals.
